# Improved Auditory Function Caused by Music Versus Foreign Language Training at School Age: Is There a Difference?

**DOI:** 10.1093/cercor/bhab194

**Published:** 2021-07-16

**Authors:** Mari Tervaniemi, Vesa Putkinen, Peixin Nie, Cuicui Wang, Bin Du, Jing Lu, Shuting Li, Benjamin Ultan Cowley, Tuisku Tammi, Sha Tao

**Affiliations:** 1 Cicero Learning, Faculty of Educational Sciences, University of Helsinki, Helsinki, Finland; 2 Cognitive Brain Research Unit, Faculty of Medicine, University of Helsinki, Helsinki, Finland; 3 Advanced Innovation Center for Future Education, Beijing Normal University, Beijing, China; 4 Turku PET Centre, University of Turku, Turku, Finland; 5 State Key Laboratory of Cognitive Neuroscience and Learning and IDG/McGovern Institute for Brain Research, Beijing Normal University, Beijing, China; 6 Faculty of Educational Sciences, University of Helsinki, Finland; 7 Cognitive Science, Department of Digital Humanities, Faculty of Arts, University of Helsinki, Finland

**Keywords:** brain development, language, learning, music, transfer

## Abstract

In adults, music and speech share many neurocognitive functions, but how do they interact in a developing brain? We compared the effects of music and foreign language training on auditory neurocognition in Chinese children aged 8–11 years. We delivered group-based training programs in music and foreign language using a randomized controlled trial. A passive control group was also included. Before and after these year-long extracurricular programs, auditory event-related potentials were recorded (*n* = 123 and 85 before and after the program, respectively). Through these recordings, we probed early auditory predictive brain processes.

To our surprise, the language program facilitated the children’s early auditory predictive brain processes significantly more than did the music program. This facilitation was most evident in pitch encoding when the experimental paradigm was musically relevant. When these processes were probed by a paradigm more focused on basic sound features, we found early predictive pitch encoding to be facilitated by music training.

Thus, a foreign language program is able to foster auditory and music neurocognition, at least in tonal language speakers, in a manner comparable to that by a music program. Our results support the tight coupling of musical and linguistic brain functions also in the developing brain.

## Introduction

Music and speech, key forms of human communication and interaction, share various principles, such as cognitive hierarchies from single items (i.e., a sound or phoneme) to complex phrases (i.e., melodies or sentences). In addition, both undergo neural processing along the auditory pathways, in the auditory cortex, and beyond (Peretz et al. 2015). Not surprisingly, interplay between learning effects in the music and speech domains has been suggested in various age groups using various empirical paradigms.

First, musical expertise and exposure facilitate several language functions. These transfer effects have been shown in cross-sectional studies, e.g., in foreign language pronunciation (Milovanov and Tervaniemi 2011), phoneme and word processing accuracy (Anvari et al. 2002; Strait et al. 2014), prosody perception (Lima and Castro 2011), and reading skills in a foreign language (Foncubierta et al. 2020; Wang et al. 2020). Musical expertise has also been shown to facilitate behavioral and neural learning efficacy of phonemes and words (Dittinger et al. 2018; Dittinger et al. 2020) across various age groups from childhood until elderly adulthood (Dittinger et al. 2019). Last but not least, causal evidence for the reciprocal relationship between music and language skills has been provided by several music intervention studies. This evidence includes (but is not limited to) EEG measures of speech sound processing (Kraus et al. 2014; Carpentier et al. 2016; Zhao and Kuhl 2016), speech-sound segmentation (François et al. 2013), reading skills (Moreno et al. 2009; Kraus et al. 2014; Nan et al. 2018), neural pitch discrimination of speech sounds (Besson et al. 2007; Moreno et al. 2009; Chobert et al. 2014) as well as phonological processing and vocabulary (Linnavalli et al. 2018).

Further, one’s native language background interacts with the neurocognition of auditory information of various kinds. Key features of one’s native language facilitate especially sound encoding when specific acoustic features affecting the semantic meaning of a given word are under interest. For instance, adult tonal language speakers (who use pitch cues to encode meaning) outperformed non-tonal language speakers (even musicians) in behavioral music tasks (Bidelman et al. 2013) and had enhanced neural functions when compared with their non-tonal language speaking counterparts (Bidelman et al. 2011). Recently, this was demonstrated in normally hearing and also cochlear-implanted children (Deroche et al. 2019). Further, linguistic backgrounds in tonal and quantity languages had differential effects on perceptual auditory encoding in musicians.^1^ The encoding was the most accurate in relation to the acoustic feature, which is most crucial in the native language of the participants (Dawson et al. 2016, 2018). However, in Dawson et al. (2018), enhanced perceptual accuracy was not seen in the subcortical neural processing in musicians.

One influential framework for explaining this transfer particularly from music to speech functions was offered by Patel (2011, 2014) in his *OPERA* hypothesis. There, emphasis is given to the anatomical *Overlap* between speech and music networks in the brain, the *Precision* needed in music encoding, the positive *Emotion* caused by music, and the *Repetition* and *Attention* required in musical practice. When these are present in music activities, a transfer from music to speech functions is likely to occur.

Based on above, we conclude that both music and language learning modulate auditory perceptual and neural functions. However, previous research has not systematically investigated whether music and language learning yield similar or different outcomes in a longitudinal program in school-aged children. In the current study, we compared the effects of music and foreign language learning on neural auditory processes in children. We tailored two group-based training programs—one in music and another in English—delivered twice a week to Chinese children aged 8–11 years, using a randomized controlled trial (RCT). We adopted an RCT paradigm because it has not yet been widely used in longitudinal intervention studies of this kind for practical reasons.

**Figure 1:**
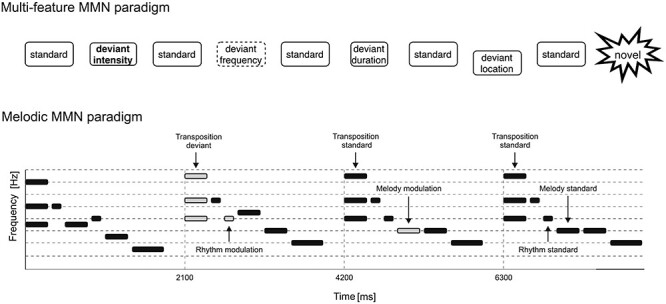
Schematic illustrations of the multi-feature paradigm (top) and of the melodic paradigm (bottom). In the multi-feature paradigm, there were four different types of deviants—duration, frequency, intensity, and location—and additional novel sounds. These were interspersed with the standard tones, with every second tone being standard and the alternating tones being either deviant or novel. In the Melodic paradigm, there were five different deviants embedded in the melody: mistuning, melody contour, rhythm, key, and timbre (bottom). These were introduced in the melody either sporadically (e.g., for mistuning and timbre) or so that they changed the continuation of the melody (e.g., for melody contour, rhythm, and key).

Before and after this one-year extracurricular program, the children participated in auditory event-related potential (ERP) recordings in two mismatch negativity (MMN) paradigms. MMN is a probe for the accuracy of the auditory cortex in encoding and predicting the content of sound sequences while a participant is not attending to the sounds (Kujala et al. 2007; Carbajal and Malmierca 2018; Fitzgerald and Todd 2020). If the sound stream contains a sound that is acoustically widely different from the majority of the sounds, a P3a response (reflecting a switch of involuntary attention toward a sounds) can follow the MMN (Gumenyuk et al. 2004; Wetzel and Schröger 2014). One of the paradigms probed the basic early sensory predictive and involuntary attentional processes in *Multi-feature MMN paradigm* (Fig. 1, top)—while the other probed early sensory-predictive processes—the musically more relevant *Melodic MMN paradigm* (Fig. 1, bottom). Our hypotheses were that the music program would facilitate neural auditory processes in both paradigms, while the foreign language program would be more specific and facilitate neural auditory processes in the multi-feature paradigm only.

## Method and Materials

### Participants

A total of 119 children between 8 and 11 years of age were recruited in an elementary school in Beijing. They were randomly assigned to English (*n* = 60) and music (*n* = 59) programs. Of these, 19 (14 boys) in the music program and 7 (3 boys) in the English program were not able to attend the programs because of overlapping schedules with other extracurricular activities. These 26 children, along with 11 newly recruited children from the same school, became members of the passive control group (*n* = 37). Of all the children, 3 in the music program, 3 in the English program and 1 in the passive control group dropped out because of being unwilling to participate in the pre-program tests. As a result, there were 123 participants at the baseline stage, enrolled in the English program (*n* = 50; mean age = 8.45, standard deviation [SD] = 0.80), music program (*n* = 37; mean age = 8.80, SD = 0.78), or control group (*n* = 36; mean age = 8.56, SD = 0.81). All the participants were native Chinese speakers.

During the program, 9 children in the English program and 7 in the music program failed to continue because the child changed schools (1), had health problems (1), or had other activities or studies at the time the program was conducted (14).

For the post-program tests, 4 children in the English program, 1 in the music program, and 18 in the control group failed to complete electroencephalogram (EEG) recordings. So, in the post-program test, 85 children completed the EEG recordings in the English group (*n* = 38; mean age = 9.18, SD = 0.75), music group (*n* = 29; mean age = 9.42, SD = 0.8), and control group (*n* = 18; mean age = 9.44, SD = 0.87).

Parents provided written informed consent and were compensated for their local transportation fees and time. The children were given small gifts, such as pens, erasers, and stickers, in appreciation of their participation. The present study was approved by the Institutional Review Board at the State Key Laboratory of Cognitive Neuroscience and Learning, Beijing Normal University, and it was conducted according to the Declaration of Helsinki.

### Content of the Training Programs

The programs lasted for two semesters, during which the children received lessons twice per week, totaling 50 sessions, in music or English after their daily curricula at their own schools. Each training session lasted 1 hour, with a 10-min break in the middle. Games, group activities, and individual hands-on activities were used to deliver the training. At the last session of each semester, there was a “Harvest Festival” held in each class to motivate the children’s learning in the classes. Children who had studied carefully or performed well in the classroom over the past semester were rewarded in the “Festival.”

The curriculum of the music program used a combination of the Kodály method and a well-established curriculum in the basic knowledge of music: Music Theory and Solfeggio, written by the Central Conservatory of Music in China (Zhao 2017). The learning content included fundamental rhythm and pitch skills, note reading, and singing. The English training program, as a second language training, focused on word decoding, phoneme awareness, letter-sound knowledge, and vocabulary from book reading. The teaching materials included the following relevant textbooks: *Letter Land* (Wendon 2009; Holt 2011), *Root Phonics English* (Sun and Lytton 2010), and *Pandeng English* (Pandeng English Project Team of State Key Laboratory of Cognitive Neuroscience and Learning in Beijing Normal University 2012).

Teachers with professional education at the master’s level in music and English language education were specifically hired for this project. In each class, there were always two teachers present: a main teacher giving the lesson and an assistant teacher helping with classroom management and assisting children who had difficulties in learning. The teachers were also given pre-program training and online support during the program. A research assistant collected checklists related to the syllabus and class implementation from the assistant teacher after each session to ensure the teaching content requirements and goals had been fully met. Each session was recorded by either video or audio for a fidelity check.

Children’s attendance at each training session of their respective programs was documented. For the music group, the mean attendance rate was 81.4%, and for the English group it was 82.5%, with no statistically significant group difference (independent t test; *t* (85) = 0.216; *P* = 0.830; Cohen’s *d* = 0.047).

After the programs were completed, we also asked the children to indicate, using a 5-point Likert scale, whether they liked the sessions (1 = *I hate it*; 2 = *I don’t like it*; 3 = *I don’t know*; 4 = *I like it a bit*; 5 = *I like it very much*). For the music group, the mean score for the question, “Did you generally like the sessions?” was 4.3, and for the English group it was 4.7, with no significant group difference (independent t-test; *t* (56.24) = 1.83; *P* = 0.072; Cohen’s *d* = 0.488; Cohen’s *d* is here used as a measure of the effect size).

However, for the more specific questions, children in the music group indicated that they liked their classes more than did children in the English group. This was shown through their opinions on the teaching content (means: 4.7 and 4.1, for the music and English groups, respectively; *t* (56.61) = 2.50, *P* = 0.015, Cohen’s *d* = 0.664) and on the ways of learning (means: 4.8 and 4.2, respectively; *t* (48.90) = 2.40, *P* = 0.020, Cohen’s *d* = 0.687).

### Data Collection Procedure

Data collection was conducted before and after the training programs’ implementation. It consisted of an individual laboratory session conducted in the EEG laboratory of the State Key Laboratory for Cognitive Neuroscience and Learning, Beijing Normal University, and a group assessment conducted in the local school. The laboratory test consisted of an EEG recording and behavioral test, both of which lasted two hours, including a rest time every half an hour (see next paragraph). During the laboratory test of the children, the parents were asked to fill in a demographic questionnaire (see below) and an additional parenting-style questionnaire (to be reported elsewhere). In addition to the current paradigms, the EEG testing procedure included paradigms probing attentional and audio-visual processes related to reading. The group assessment included English reading performance, phonological awareness, mathematical skills as well as motivational questionnaires. These results will be reported elsewhere.

### Behavioral Measurements

Three subtests were chosen from the Wechsler Intelligence Scale for Children (WISC) test (Wechsler 2003)—block design, digit span and vocabulary—to obtain a cognitive profile of the children and to determine whether the music and language programs had any effects on these cognitive functions. Group-based tests on learning achievements were also conducted. The longitudinal data of these cognitive measures will be reported elsewhere.

The demographic information collected included children’s gender and age, parents’ age, and family socioeconomic status (SES). To measure family SES, we collected the educational level of both mothers and fathers, from no education to the doctoral level. Here, we report the SES based on the family annual income, which was also asked to be reported by the parents using the categories 0–3000 Chinese renminbi (RMB), 3000–5999 RMB, 6000–9999 RMB, 10000–29 999 RMB, 30000–49 999 RMB, 50000–99 999 RMB, 100000–149 999 RMB, 150000–200 000 RMB, and more than 200 000 RMB (100 RMB roughly equaling 15 United States dollars at the time of writing). These income values were analyzed by assigning to each respondent the median RMB value of their category (250,000 RMB for the highest category).

There were no significant differences among the three groups before the training programs in terms of age, SES (annual income of the parents), or intelligence quotient (IQ; pooled across the WISC subtests block design, digit span, and vocabulary). This is shown by statistical comparisons for age (*F*[2, 120] = 2.806; *P* = 0.129; *η2* = 0.034), for SES (*F*[2, 118] = 0.669; *P* = 0.514; *η2* = 0.011); and for IQ (*F*[2, 120] = 1.801; *P* = 0.169; *η2* = 0.029; *η2* is here reported as a measure of the effect size).

The proportion of gender differed significantly among the groups (*X2*[2, *N* = 123] = 15.271; *P* = 0.0005). This was caused by the presence of more boys in the English than in the music group (see Tables 1 and 5). It is of note that after random group allocation, there were 26 participants who did not join the experimental (Music, Language) groups but rather were became participants of the passive control group, 19 of them being boys.

### ERP Paradigms

#### Multi-Feature Paradigm

The multi-feature paradigm was delivered via headphones when the participants were instructed to watch a silent movie. In this procedure, their brain’s ability to encode and predict the sound sequences was probed without the involvement of their attentional resources. In this multi-feature paradigm, four different tones were delivered in the context of a standard tone. These tones were presented in an alternating order, with the standard tones in a pseudorandom manner, so that two successive deviant tones were never from the same category (Fig. 1). Previously, the multi-feature paradigm has been shown to elicit MMN responses with amplitude and latency corresponding to MMN responses in the traditional oddball paradigm but in a considerably shorter time (Pakarinen et al. 2010). It has been used among various age groups, including pre-term infants (Francois et al. 2020; Kostilainen et al. 2020) and pre-school children (Lovio et al. 2009; Kuuluvainen et al. 2016; Linnavalli et al. 2018), as well as among healthy (Honbolygó et al. 2017) and clinical (Gürses et al. 2020) adult populations.

The standard tones (*P* = 0.50, *n* = 1200) had a fundamental frequency of 500 Hz, were 100 ms in duration (including 5-ms rise and fall times), and were presented binaurally via headphones. They included the first two harmonics, which were − 3 and − 6 dB in intensity compared to the fundamental, respectively. The harmonics were included in the stimulation in order to increase the amplitude of the MMN (Tervaniemi et al. 2000a, 2000b).

The deviant tones (*P* = 0.10, *n* = 120/deviant type) differed from the standard tones in one of four features but were otherwise identical to the standard tones. The frequency deviants had a fundamental frequency of 450 or 550 Hz, the duration deviants were 65 ms in duration, the intensity deviants were − 5 dB compared to the standard, and the location deviants were presented only from the left or right headphone. The sound sequences also included novel sounds, such as a dog barking or car driving (*P* = 0.10). In prior studies, the gap deviant has also been used, but due to suboptimal responses, this type of deviant sound was omitted from the current paradigm. The stimuli were presented with a stimulus onset asynchrony of 500 ms, and the duration of the sequence was 10 min.

#### Melodic MMN Paradigm

We employed an adapted version of the Melodic MMN paradigm, which has been used in previous studies among pre-school children (Putkinen et al. 2019), school-aged children (Putkinen et al. 2014), and adults (Tervaniemi et al. 2014, 2016). The paradigm was composed of 360 piano melodies of 2.1 s in duration each. The F0 of the tones varied between 233.1 and 466.2 Hz. Each melody consisted of six sounds: a 300-ms major triad chord followed by two 125-ms tones (short inter-tones) and two 300-ms tones (long inter-tones) in varying order, and a 575-ms tonic tone at the end of the melody (end tone). The inter-stimulus interval between the tones was 50 ms, and the silent interval between the melodies was 125 ms. For illustration, see Figure 1.

**Table 1:** Background information (age, gender, SES, and attendance rate) of the participants

Demographic statistics						
	Music group		English group		Control group	
	Pretest (*n* = 37)	Posttest (*n* = 29)	Pretest (*n* = 50)	Posttest (*n* = 37)	Pretest (*n* = 36)	Posttest (*n* = 18)
Age (M ± SD)	8.80 ± 0.78	9.41 ± 0.80	8.45 ± 0.80	9.22 ± 0.73	8.56 ± 0.81	9.44 ± 0.87
Gender	*n*(boys) = 9	*n*(boys) = 6	*n*(boys) = 26	*n*(boys) = 21	*n*(boys) = 25	*n*(boys) = 12
	*n*(girls) = 28	*n*(girls) = 23	*n*(girls) = 24	*n*(girls) = 16	*n*(girls) = 11	*n*(girls) = 6
SES (M ± SD)	121 378 ± 69 360	112 966 ± 65 054	99 408 ± 81 000	98 824 ± 79 000	124 722 ± 69 312	118 056 ± 80 916
Attendance rate (%)	Mean ± SD = 88.76 ± 15.81 Median = 96 Mode = 98 Maximum = 100 Minimum = 38		Mean ± SD = 88.76 ± 17.81 Median = 96 Mode = 100 Maximum = 100 Minimum = 24		—	

Melodic MMN paradigm included the following deviants: 1) melody modulation (one of the long inter-tones was replaced with another in-key tone), 2) rhythm modulation (the rhythmic pattern was modulated by switching the durations of two inter-tones), 3) transposition (the melody was transposed up or down by one semitone), 4) timbre (a long inter-tone or the final tone was played with a flute timbre instead of a standard piano timbre), and 5) mistuning (a long inter-tone was mistuned by half a semitone). The duration of the paradigm was 13 min. In prior studies, a timing delay has also been used, but due to suboptimal responses observed after that deviant, it was omitted from the current paradigm.

Like the multi-feature paradigm, the Melodic paradigm was delivered via headphones while the participants watched a silent movie.

### E‌EG Recording and Analyses Statistical Analysis

The EEG recording was conducted using 128-channel HydroCel Geodesic Sensor Net (Electrical Geodesics, Inc., Eugene, OR, USA). The filter bandwidth was 0.1–100 Hz, and the sample rate was 1000. Cz was used as the online reference electrode during the recordings. The individual electrode impedance was kept below 50 kΩ (Tucker 1993).

#### E‌EG Data Processing

The EEG data were preprocessed using MATLAB R2018b with EEGLAB v14.1.2b (Delorme and Makeig 2004) and Computational Testing for Automated Preprocessing (CTAP; Cowley et al. 2017; Cowley and Korpela 2018) toolboxes. The data were first downsampled to 500 Hz and filtered offline with a bandpass filter of 0.5–30 Hz. Signals were re-referenced to the average of the mastoids for artifact detection and further analysis. Bad channels were rejected manually and then interpolated. Bad segments were rejected if 15% or more of the channels had an absolute amplitude higher than 150 μV. Independent component analysis was conducted, and the Fully Automated Statistical Thresholding for EEG Artifact Rejection (FASTER) plugin (Nolan et al. 2010) was used to detect and remove artifactual components. The recordings were segmented into epochs 50 ms prior and 500 ms after the stimulus onset, and the data was downsampled to 250 Hz for further analysis. Epochs with amplitude changes exceeding ±100 μV were discarded. The remaining trials were averaged separately for each deviant and standard.

For each participant, difference waves (i.e., deviant minus standard) were computed using R version 3.6.2. From these difference waves, the mean amplitudes were calculated at Fz for each deviant over a 50-ms time window centered on the latency of the most negative peak between 130 and 250 ms (MMN) or the most positive peak between 200 and 300 ms (P3a). To reduce high-frequency noise, the average across the channels F3, Fz, and F4 was used in subsequent analyses.

### Statistical Analysis

The effect of language and music training programs on the MMN amplitude was analyzed in both paradigms with a linear mixed model (LMM) using R package *lme4*, and *P* values were computed with the lmerTest package, using Satterthwaite’s method to estimate degrees of freedom. In the model, group (control, English, or music) and time (pre- or post-intervention) were used as fixed factors, and participant was used as a random intercept factor. In this way, random effects resulting from repeated measures on the same participants were accounted for. The estimated marginal means were computed using the emmeans package and Bonferroni-corrected post hoc comparisons were performed where statistically significant effects were seen in the LMM.

**Figure 2:**
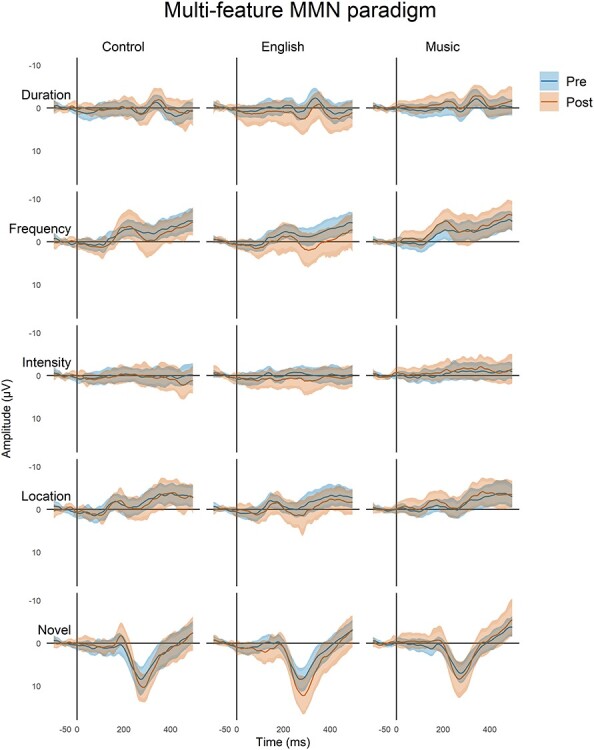
Deviance responses for the multi-feature paradigm in three groups of participants for the four deviants and the novel sounds (F3, Fz, and F4 data pooled together). Each plot shows the mean responses (solid lines) of the pre-program (blue) and post-program (brown) recordings. These mean responses are surrounded by two bands: the naïve 95% confidence intervals (CIs) of all the time points (lighter, narrower filled curves), and the MWE confidence bands of the time series (darker, wider filled curves; see Materials and Methods).

**Figure 3:**
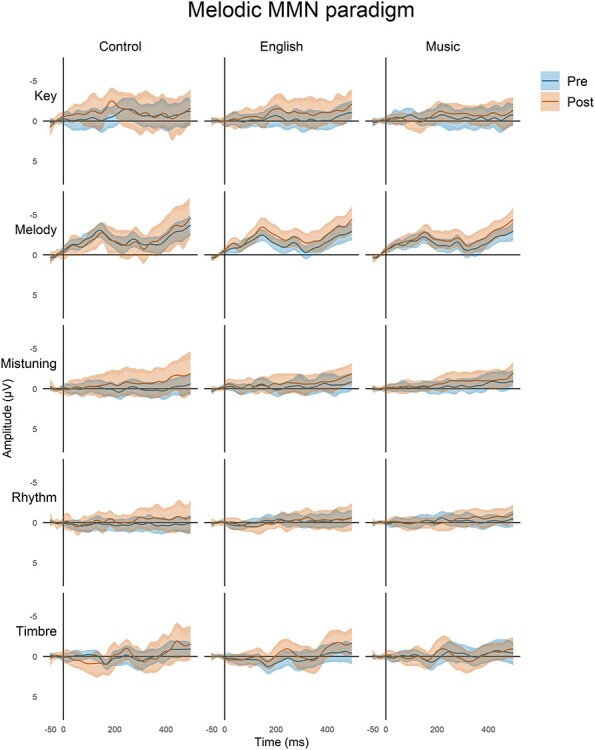
Deviance responses for the Melodic paradigm in three groups of participants for five deviants (F3, Fz, and F4 data pooled together). Each plot shows the mean responses (solid lines) of the pre-program (blue) and post-program (brown) recordings. The mean responses are surrounded by two bands: the naïve 95% CIs of all time points (lighter, narrower filled curves), and the MWE confidence bands of the time series (darker, wider filled curves; see Materials and Methods).

In Figures 2 and 3, we visualize the data using minimum-width envelopes (MWEs), developed by Korpela et al. (2014). These generalize univariate confidence intervals (CIs) to multi-variate time series data. MWE bands tend to be wider than CIs because they account for the non-independent nature of time series data, yet they allow a similar visual interpretation of the data because the true average of the distribution traverses inside the lower and upper bounds with a probability of 1 − α (where α is the desired level of control of the Type I error). The MWE model thus represents a statistical test of whether samples from two conditions are drawn from separate distributions: if at any point, the mean of one sample is outside the MWE of the other, it shows that the curves as a whole are statistically significantly different. Here, we used *α* = 0.05/3 to match the degree of control for multiple comparisons applied in the main analysis. Although the MWEs provide a visual significance test, they do not differentiate separate components of the ERP, and thus, our reporting focuses on the components derived using the standard approach.

## Results

In brief, the results did not support our hypotheses. Instead, they showed that in the Melodic paradigm (probing musically relevant sound features), the language-training program facilitated the basic auditory processing more than the Music program did. In the multi-feature paradigm, the music program significantly facilitated the basic processing of pitch (sound frequency) information. Within both paradigms, the participants were instructed to concentrate on watching a silent movie and to ignore the sounds. This suggests that the neural processes modulated by the programs do not depend on the attentive listening skills of the children.

### Multi-Feature Paradigm

In total, there were four different deviants embedded in this paradigm: duration, frequency, intensity, location, and additional novel sounds (Fig. 1, top). As displayed in Figure 2, all deviant and novel sounds evoked MMN and/or P3a *except* the intensity deviant (for ERPs, see Supplementary Material). The mean amplitudes and latencies for the MMN and P3a responses obtained in the multi-feature paradigm are listed in Table 2, and the results of the linear mixed-model analyses of the amplitudes are listed in Table 3.

**Table 2:** MMN and P3a amplitudes (μV) at *F*z and latencies (ms) for the Control, English, and Music groups before and after intervention in the multi-feature paradigm (mean and SD)

	Control		English		Music	
	Pre	Post	Pre	Post	Pre	Post
Frequency MMN						
Amplitude	−1.94 (1.63)	−2.16 (2.07)	−1.57 (1.81)	−1.23 (2.64)	−1.73 (2.30)	−2.91 (2.03)
Latency	210.12 (32.72)	208.00 (27.58)	210.86 (29.55)	203.67 (34.00)	208.78 (30.51)	207.20 (29.10)
Frequency P3a						
Amplitude	−0.98 (1.90)	−0.30 (2.21)	−0.56 (2.19)	0.62 (2.96)	−1.15 (2.12)	−1.13 (2.58)
Latency	301.41 (43.45)	282.53 (45.34)	304.98 (42.38)	296.44 (45.40)	300.56 (39.07)	303.20 (43.82)
Location MMN						
Amplitude	−1.25 (1.64)	−1.46 (1.36)	−1.33 (1.59)	−1.19 (2.13)	−0.90 (1.29)	−1.41 (1.77)
Latency	171.06 (32.02)	170.53 (27.75)	160.98 (31.03)	166.00 (33.02)	171.56 (39.12)	166.08 (28.48)
Location P3a						
Amplitude	−0.32 (1.77)	−0.32 (1.72)	−0.08 (1.90)	0.52 (2.69)	−0.18 (1.90)	−0.30 (1.96)
Latency	268.12 (39.31)	244.84 (45.66)	257.80 (38.82)	244.00 (42.87)	262.89 (40.31)	258.24 (41.30)
Duration MMN						
Amplitude	−0.38 (1.49)	−0.43 (1.29)	−0.62 (1.83)	−0.21 (2.59)	−0.50 (1.63)	−1.09 (1.68)
Latency	203.06 (36.14)	204.63 (36.13)	207.76 (38.97)	203.89 (35.92)	210.00 (40.86)	212.48 (32.42)
Duration P3a						
Amplitude	0.34 (1.48)	0.02 (1.65)	0.09 (2.29)	0.92 (2.86)	0.10 (2.04)	−0.11 (1.77)
Latency	284.82 (40.30)	289.89 (42.09)	281.96 (44.33)	282.33 (43.69)	290.78 (38.72)	275.84 (43.81)
Intensity MMN						
Amplitude	−0.84 (1.24)	−0.83 (1.29)	−1.08 (1.41)	−0.62 (1.97)	−1.05 (1.79)	−1.19 (1.81)
Latency	205.18 (60.05)	221.89 (62.43)	221.47 (60.19)	222.78 (60.25)	222.22 (65.88)	247.84 (52.01)
Novel MMN						
Amplitude	−0.27 (1.78)	−0.81 (1.57)	−0.58 (1.62)	−0.21 (2.55)	−0.06 (1.49)	−0.83 (1.52)
Latency	164.00 (28.03)	175.37 (22.52)	165.8 (28.71)	177.56 (26.81)	166.11 (32.89)	168.32 (26.56)
Novel P3a						
Amplitude	3.69 (2.75)	4.54 (2.44)	3.84 (2.77)	5.62 (3.89)	2.88 (2.29)	3.60 (3.12)
Latency	307.29 (57.43)	297.89 (64.75)	299.27 (59.06)	283.89 (63.45)	310.78 (57.47)	319.68 (54.73)

**Table 3:** Results of linear mixed models for multi-feature paradigm

	Time			Group			Time × Group interaction		
	*d*f	*F*	*P*	*d*f	*F*	*P*	*d*f	*F*	*P*
Frequency MMN	111.75	1.43	.235	129.19	3.15	**.046**	110.61	3.32	**.040**
Frequency P3a	110.79	3.34	.070	129.70	3.53	**.032**	109.68	1.38	.256
Location MMN	114.86	0.66	.417	120.82	0.19	.824	113.44	0.74	.480
Location P3a	110.15	0.29	.593	126.22	1.17	.315	108.97	0.85	.431
Duration MMN	117.93	0.18	.675	124.67	0.85	.431	116.56	1.35	.264
Duration P3a	110.82	0.02	.901	121.30	0.74	.479	109.49	1.76	.176
Intensity MMN	112.78	0.20	.659	126.15	0.62	.540	111.54	0.85	.432
Novel MMN	112.21	1.51	.222	122.06	0.08	.920	110.88	2.19	.117
Novel P3a	113.10	6.46	**.012**	123.53	3.69	**.028**	111.79	0.50	.608

Note: Significant *P*-values (< 0.05 after Bonferroni correction) are marked in bold.

#### Frequency Deviant

We observed a main effect of group (i.e., music vs. language vs. passive control) and of the interaction between group and time in the frequency MMN amplitude (Table 3).

Regarding the main effect of group, the post hoc tests did not reveal any significant differences in paired group comparisons (all *P* values > 0.05). Regarding the interaction between group and time, post hoc tests revealed that it resulted from a larger MMN in the Music group than in English group in post-training recordings (*P* < 0.01, Cohen’s *d* = 0.957) as well as from a larger MMN in post-training (compared pre-training) recordings in the Music group (*P* < 0.05, Cohen’s *d* = 0.670).

In the P3a amplitude, there was a main effect of group (Table 3). According to post hoc tests, this was caused the English group having larger P3a than the Music group (*P* < 0.05, Cohen’s *d* = 0.573).

#### Novel Sounds

In the P3a amplitude elicited by novel sounds, there were main effects of time and group (Table 3). The main effect of time resulted from P3a becoming larger in amplitude from the pre to post-program recordings. The main effect of group resulted from a larger P3a amplitude in the English group than in the Music group (*P* < 0.05, Cohen’s *d* = 0.526).

#### Location, Duration, and Intensity Deviants

Regarding MMN and P3a responses related to location, duration, and intensity, there were no statistically significant main effects of group or time or their significant interactions (see Table 3).

### Melodic MMN Paradigm

In total, there were five different deviants embedded in the melody: mistuning, melody contour, rhythm, key, and timbre (Fig. 1, bottom). Difference waves for each deviant are shown in Figure 3 illustrating the MMN to melody, key, and timbre deviants (for ERPs, see Supplementary Material). The amplitudes and latencies for each deviant are summarized in Table 4 and the outcomes of the training programs are illustrated by the LMM analyses in Table 5.

**Table 4:** MMN amplitudes (μV) at *F*z and latencies (ms) for the Control, English and Music groups before and after the training program (mean (SD)) in the Melodic paradigm

	Control		English		Music	
	Pre	Post	Pre	Post	Pre	Post
Melody contour						
Amplitude	−2.80 (1.57)	−2.47 (1.74)	−2.28 (1.36)	−3.18 (2.06)	−2.06 (1.53)	−2.58 (1.42)
Latency	170.74 (39.27)	166.67 (31.73)	178.96 (41.02)	169.51 (32.43)	169.95 (35.04)	177.24 (36.38)
Mistuning						
Amplitude	−0.02 (1.65)	−0.71 (2.33)	−0.21 (1.63)	−0.71 (2.17)	−0.26 (1.58)	−0.89 (1.75)
Latency	211.54 (36.36)	212.44 (33.63)	208.32 (35.66)	214.70 (30.78)	200.32 (36.58)	213.38 (32.90)
Rhythm						
Amplitude	0.21 (1.40)	−0.28 (1.29)	−0.34 (1.30)	−0.13 (1.66)	−0.43 (1.34)	−0.05 (1.69)
Latency	195.89 (32.33)	184.89 (29.82)	192.64 (31.00)	194.59 (32.11)	197.41 (28.38)	201.38 (29.13)
Key						
Amplitude	−1.37 (2.64)	−2.11 (2.02)	−0.56 (2.68)	−1.45 (2.85)	−0.65 (2.28)	−1.07 (1.87)
Latency	212.00 (31.12)	192.22 (29.34)	194.24 (29.11)	203.89 (23.82)	202.33 (30.93)	207.86 (26.97)
Timbre						
Amplitude	−0.02 (1.98)	−0.71 (1.38)	0.36 (2.07)	−0.80 (2.10)	−0.46 (2.30)	−0.82 (2.23)
Latency	221.60 (32.30)	228.22 (24.37)	212.48 (37.51)	217.08 (33.03)	219.35 (36.79)	215.86 (26.82)

**Table 5:** Results of linear mixed models where MMN responses were predicted by time and group

	Time			Group			Time × Group interaction		
	*d*f	*F*	*P*	*d*f	*F*	*P*	*d*f	*F*	*P*
Response									
Melody contour	114.29	2.22	.139	126.31	0.89	.411	112.76	3.22	**.044**
Mistuning	113.33	7.02	**.009**	128.52	0.17	.844	111.86	0.03	.975
Rhythm	113.97	0.05	.819	118.08	0.36	.701	112.35	1.49	.230
Key	104.51	3.58	.061	105.94	1.77	.176	102.85	0.16	.849
Timbre	111.33	6.17	**.015**	127.50	0.65	.525	109.87	1.23	.296

Note: Significant *P*-values (<0.05; after Bonferroni correction) written in bold.

In the melody contour deviant, there was a significant interaction between group and time. Post hoc pairwise comparisons revealed that this resulted from a larger MMN amplitude in post- than pre-training measurements in the English group, but not in the other groups (Cohen’s *d* = 0.627, *P* < 0.01). In the mistuning and timbre deviants, there was a significant main effect of time, caused by a larger MMN amplitude in post- than pre-training measurements across the groups (Cohen’s *d* mistuning = 0.405 and Cohen’s *d* timbre = 0.429; both *P* < 0.05). In the MMN elicited by rhythm and key deviants, there were no statistically significant differences.

## Discussion

Our study compared the effects of music and foreign language training programs on neural sound discrimination processes in school children using a longitudinal RCT. Using two advanced auditory ERP paradigms before and after our one-year programs, we probed the early sensory-predictive auditory processes that are activated even when voluntary attention is not given to sound sequences. Additionally, by including acoustically novel sounds in one of the paradigms, we were able to determine the impact of the training programs on involuntary attention.

In opposition to our hypotheses, the extracurricular group-based program in a foreign language (i.e., English in Chinese children) facilitated the children’s early sensory-predictive processes in the auditory modality significantly more than did the program in music, particularly when the experimental paradigm was musically relevant. When these processes were probed by a paradigm more focused on basic sound features, we found the music program to facilitate sensory-predictive pitch encoding more than the language program did. So, our findings are most surprising when they concern the promises of foreign language learning to foster the encoding of musical features, as probed here by the melodic MMN paradigm. Thus, in brief, our findings provide novel insight about the reciprocal capacity of the auditory brain circuits to optimize and facilitate functions for music and speech by tuition in one of these domains.

Anatomically, long-term expertise in music (versus speech/language) depends on shared as well as separable brain areas, depending on the level of the cognitive processes involved. The music-syntactic expectancy violations introduced within well-structured excerpts of classical Western music are known to activate Broca’s area (which is also involved in language-syntactic processing; Maess et al. 2001). In contrast, the early sensory processing of deviances activates different parts of the thalamus in terms of musical versus speech sounds (Tervaniemi et al. 2006b).

Functionally, the interplay between long-term high-level expertise in music versus speech was evidenced three decades ago: a high-level of expertise in music was shown to modulate attentionally driven auditory processes in language as well (Besson et al. 1994). Corresponding evidence was obtained through a pre-attentively evoked MMN response (Martínez-Montes et al. 2013) and brain-stem-driven frequency-following response recordings (Strait et al. 2014). Importantly, the impact of musical expertise has been recently found in novel word learning tasks both behaviorally and neurally (Dittinger et al. 2018, 2019, 2020).

In the current study, for the first time, we obtain causal evidence of the impact of a group-based foreign language training program to facilitate neural auditory processing more than music program. Of particular importance is the fact that the foreign language program was more influential than was music program within the musically complex stimulus paradigm, even when both training programs were given with comparable content and intensity. In parallel, it is of note that also music program facilitated pitch processing but only in a more simplified stimulus paradigm. These results are highly promising because they reveal the high degree of auditory neuroplasticity present in pre-adolescence (i.e., 8–11 years of age), which, according to some views, is later than the optimal age for auditory neuroplasticity (e.g., Wan and Schlaug 2010; for discussion see also White et al. 2013). Our results are also promising when considering the type of music and foreign language training programs used, which were group based, did not require a considerable amount of homework, and involved a maximum of 50 lessons during an academic year.

When considering the impact of the current results, we should take into account that the children were all native Mandarin Chinese speakers and thus highly sensitive to pitch of any sound, including speech in their native or non-native language. This is caused by the importance of pitch and contour in denoting semantic meaning in Mandarin Chinese. Thus, the English language program might have optimally activated their general auditory processing capacity and particularly pitch processing capacity, which is shared by music and speech information (see also Tong et al. 2018). Notably, the data indicate that this capacity was activated in the brain recordings even when the children were not attending to the sounds but were focusing their attention on a silent movie. Also, the enhanced processing capacity was not generalized to all auditory features but was restricted in both paradigms to the deviant that is spectrally transmitted and relatively complex (i.e., melody contour and pitch), as are pitch and pitch contours in the children’s native tonal language.

Methodologically, it is of note that the group allocation of the participants in the current investigation was, in the first place, random between the English and music programs for the 119 participants who were originally interested in participating in extracurricular activities. However, there were 19 participants in the music program and 7 participants in the English program who were not able to attend their primary program despite their initial interest. Therefore, they were given the option of participating in the control group. Additionally, 7 participants also originally interested in the study were not included because they were not willing to participate in the pre-training tests, and another 16 participants did not continue in the programs for various reasons (see section Materials and Methods).

This leads us to question the meaningfulness of an RCT design in a study in which the long-term effects of a given training program are under observation. In recent transfer literature, RCTs have been requested because they are considered to be more objective and, thus, of higher scientific quality than are studies in which personal choice in the completion of a given training program is a factor (e.g., see Sala and Gobet 2020). However, based on our study and other contributions, it is suggested that RCTs might not be optimal paradigms in longitudinal intervention studies in educational domains.

It is not possible to know the exact reasons for the non-interest of some child participants in the current study in continuing (or even starting) the extracurricular programs. However, the quite high drop-out rate may be an indication of a non-optimal match between the children’s interests and the content of the programs. Such a high number of participants quitting the programs leads to a situation resembling that of a personal choice study, as only the motivated participants remained in the long-lasting program (for related discussion, see Habibi et al. 2018; Tervaniemi et al. 2018).

In our view, an RCT is an optimal choice for shorter term programs or for programs involving less intensive training. The longer and/or more intensive the programs are, the more emphasis should be given to the personal choice of the participants. Then, the intrinsic motivation of the participants will be optimized (Schellenberg 2015). Moreover, if participants are given a personal choice regarding the content of the training they receive, they will be more easily engaged by it even outside the lessons, thus maximizing the impact of the training. For any training to have an impact on any neurocognitive function (be it near- or far-transfer learning), practice—or at least active engagement during the lessons—is necessary.

Another important issue regarding the RCT protocol used in the present study is that there were no other differences in the background variables or outcome measures at the pre-training stage except gender. This suggests that even if many publications in the field have reported pseudorandom allocation of their study participants to ensure the lack of pre-training differences, this was not necessary in the current study since large enough groups were recruited from a relatively homogenous pool of participants.

Finally, as mentioned in the Introduction section, the OPERA hypothesis of Patel (2011, 2014) has been used to explain the near- and far-transfer effects of music in the speech domain, one critical feature being positive emotions related to music. That said, in the current study, when we asked the children whether they generally liked their training program, the answers among students in the music and language groups did not differ; yet, for specific questions regarding their liking of the content and manner of teaching in the program, the students in the music program gave more positive remarks than did those in the language program.

At first glance, the OPERA hypothesis and the current results thus seem to be in contradiction when it comes to the role of emotions. Here, it is of note that, as denoted by prior literature, positive emotions and preferences are conceptually and neurally linked but not identical (Brattico et al. 2016). Moreover, the subjective experiences evoked by music were recently shown to differ across different cultures (Cowen et al. 2020) and, importantly, were argued to be biased because of being mediated by language (Bowling 2020). Therefore, we cannot confirm or reject Patel’s (2011, 2014) notion of the importance of emotional processes in auditory transfer functions but rather call upon future research to illuminate the interaction between emotional and neurocognitive processes in longitudinal training paradigms.

In conclusion, we provide here novel causal evidence for the assertion that the extracurricular group-based program in a foreign language applied in this study facilitated the children’s auditory predictive processes more than the music program did, even though the experimental paradigm was not linguistically relevant. When these neurocognitive processes were probed using a paradigm more focused on basic sound features, we found music training to facilitate pitch encoding. Thus, there are transfer effects in the neural functions obtained by auditory (music/language) training programs. These may be observed even at the level of pre-attentive processes, suggesting a tight coupling between musical and linguistic brain functions.

## Supplementary Material

Tervaniemi_Supplementary_Legends_bhab194

Melody_BW_ERP_bhab194

Multifeature_BW_ERP_bhab194

## Footnotes

1 In quantity languages such as Finnish, phoneme duration carries semantic meaning. This was seen as the facilitation of subcortical and cortical brain responses in Finnish participants when compared to speakers of other languages (Ylinen et al. 2006; Tervaniemi et al. 2006a; Dawson et al. 2016).
