# Supplementary material for: Improved Auditory Function Caused by Music Versus Foreign Language Training at School Age: Is There a Difference?
Source: Cereb Cortex. 2021 Jul 16;32(1):63–75. doi: 10.1093/cercor/bhab194 (PMC8634570; doi:10.1093/cercor/bhab194)
Supplement: Tervaniemi_Supplementary_Legends_bhab194 [file tervaniemi_supplementary_legends_bhab194.docx]

Supplementary Figure 1: Figure 1. Event-related potentials for the Multi-feature paradigm in three groups of participants for the four deviants and the novel sounds (F3, Fz, and F4 data pooled together). Grey lines denote pre-program recordings and black lines post-program recordings. Dashed line for the standard sounds and solid line for the deviant sounds.

Supplementary Figure 2: Figure 2. Event-related potentials for the Melodic paradigm in three groups of participants for the four deviants and the novel sounds (F3, Fz, and F4 data pooled together). Grey lines denote pre-program recordings and black lines post-program recordings. Dashed line for the standard sounds and solid line for the deviant sounds.
