# Supplementary figures and images for: Improved Auditory Function Caused by Music Versus Foreign Language Training at School Age: Is There a Difference?
Source: Cereb Cortex. 2021 Jul 16;32(1):63–75. doi: 10.1093/cercor/bhab194 (PMC8634570; doi:10.1093/cercor/bhab194)

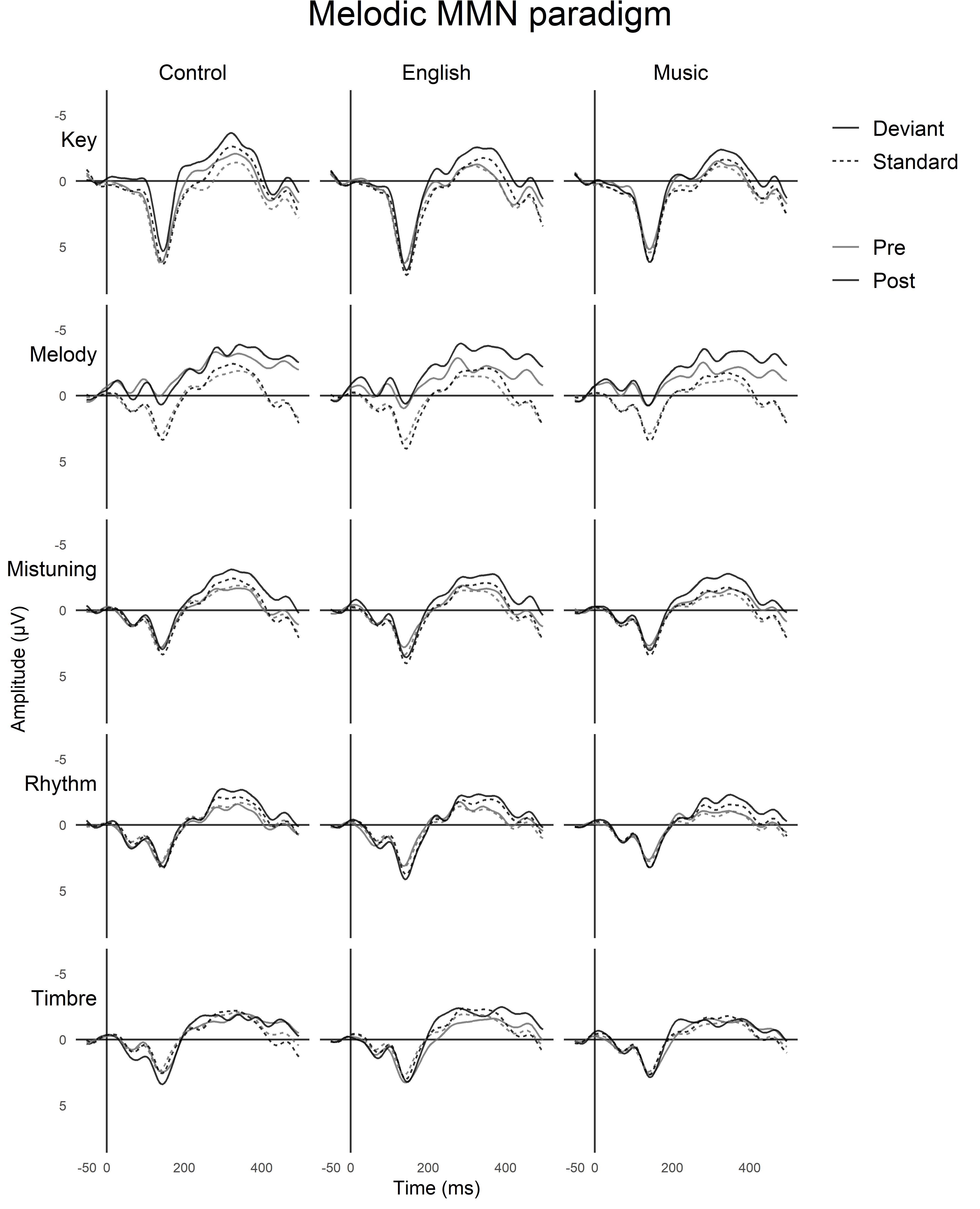

Supplement: Melody_BW_ERP_bhab194 [file melody_bw_erp_bhab194.jpeg]

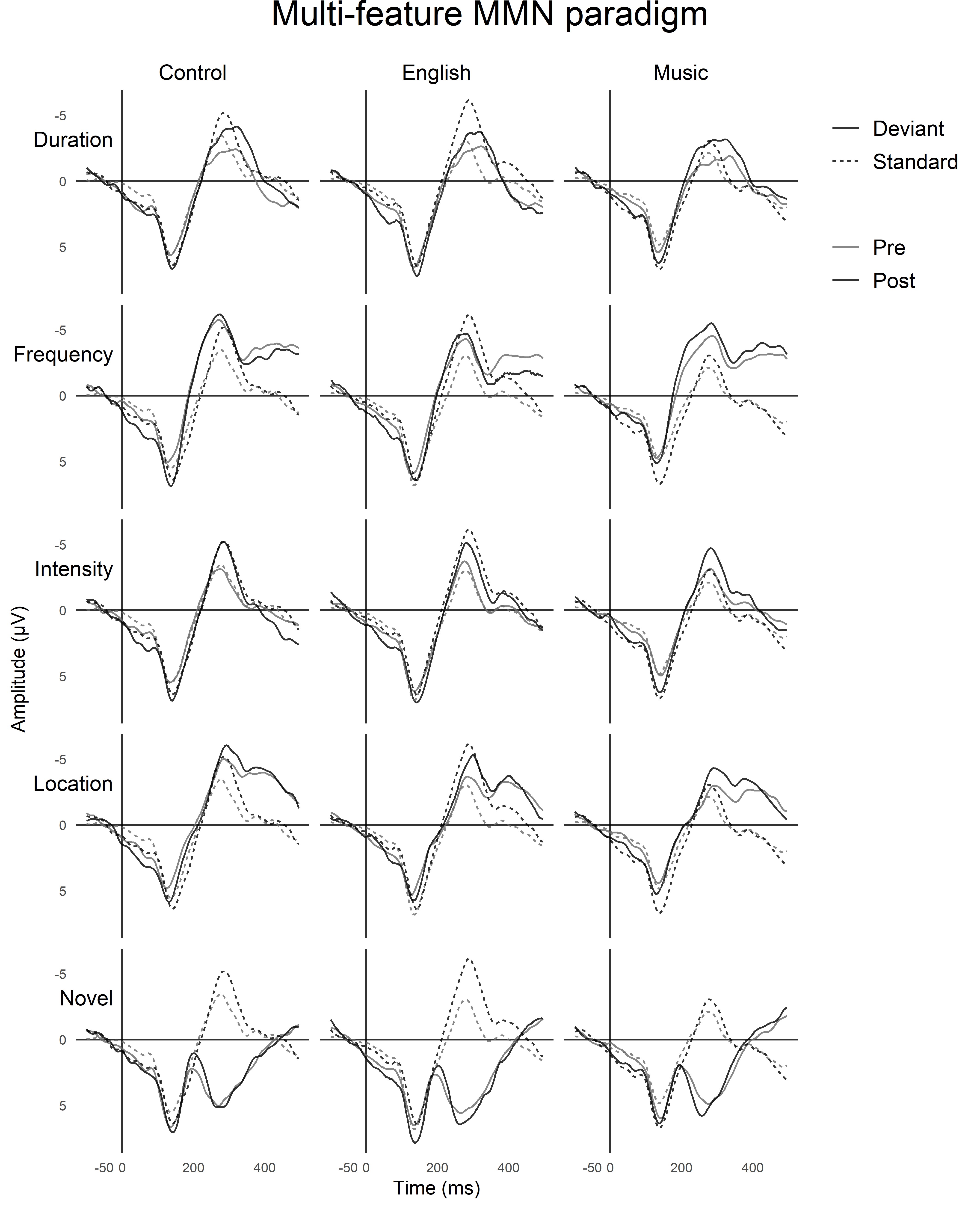

Supplement: Multifeature_BW_ERP_bhab194 [file multifeature_bw_erp_bhab194.jpeg]
